# Prevalence of self-reported early glaucoma eye drop bottle exhaustion and associated risk factors: a patient survey

**DOI:** 10.1186/1471-2415-14-79

**Published:** 2014-06-13

**Authors:** Daniel B Moore, Charlene Walton, Kristy L Moeller, Mark A Slabaugh, Raghu C Mudumbai, Philip P Chen

**Affiliations:** 1 Department of Ophthalmology, University of Washington, 325 Ninth Ave, Seattle, WA 98104-2499, USA; 2 Seattle University, Seattle, WA, USA

**Keywords:** Glaucoma, Eyedrop, Medication, Treatment, Adherence, Compliance, Visual impairment, Low vision, Blindness

## Abstract

**Background:**

One barrier to patient adherence with chronic topical glaucoma treatment is an inadequate amount of medication available between prescription refills. We examined the self-reported prevalence of early exhaustion of glaucoma eye drops prior to a scheduled refill, and associated risk factors.

**Methods:**

This cross-sectional survey was performed at a University-based clinical practice. Glaucoma patients at the University of Washington who were experienced with eye drop application and were on a steady regimen of self-administered glaucoma drops in both eyes took a survey at the time of clinic examination. The main outcome measure was self-reported early eye drop bottle exhaustion.

**Results:**

236 patients were eligible and chose to participate. In general, patients included were relatively healthy (mean 2.3 comorbid medical conditions). Sixty patients (25.4%) reported any problem with early exhaustion of eye drop bottles, and this was associated with visual acuity ≤ 20/70 in the better eye (P = .049). Twelve patients (5.1%) reported that they “often” (5–7 times per year), “usually” (8–11 times per year) or “always” ran out of eye drops prior to a scheduled refill. Patients affected by this higher level (≥5 times yearly) of eye drop bottle exhaustion were more likely to have poor visual acuity in their worse eye ≤ 20/70 (P = .015) and had significantly lower worse-eye logMAR (P = .043).

**Conclusions:**

Self-reported early glaucoma bottle exhaustion regularly affected 5% of patients in our population and 25% reported early exhaustion at least once; the main risk factor was poor vision in at least one eye. These results may not be generalizable to a broad patient population, or to those inexperienced with eye drop self-administration. However, this pilot study compels further evaluation and consideration of early eye drop bottle exhaustion in glaucoma patients.

## Background

Glaucoma, the second leading cause of global blindness [1], is a progressive optic neuropathy with characteristic structural and functional abnormalities. Several clinical trials have demonstrated that reducing intraocular pressure (IOP) slows glaucoma progression [2,3], and proper use of topical ocular antihypertensives improves outcomes. However, numerous reports suggest patients comply with only 70% or less of prescribed topical treatments [4-7].

Multiple reasons for poor adherence with topical glaucoma treatment have been implicated including situational and environmental factors, medication regimen, patient-related and provider-related factors [8]. Another barrier to patient compliance is an inadequate amount of medication available between scheduled prescription refills, because of difficulty in eye drop administration with resultant wastage. This is an issue in the United States (and may be in other developed nations) where prescription drug benefits ensure access to medication for most of the population, but also control the time interval before a refill may be obtained. This may also be of concern in less developed countries where access to and availability of medication may be limited. Although it is not uncommon in the clinic setting to hear patient complaints about the difficulty of getting refills if eye drop bottles are prematurely exhausted, no data exists in the literature regarding the prevalence of this phenomenon. Therefore we examined the prevalence of early exhaustion of glaucoma eye drops prior to a scheduled refill, and associated risk factors. Recent Centers for Medicare and Medicaid Services (CMS) changes now allow refills at 70% of the predicted days of use for patients with CMS-sponsored prescription drug coverage in the United States [9], which applies only to roughly 50% of the glaucoma patient population. As of late 2013, nine states also have passed and several others have pending legislation to provide coverage for all patients. The present study was based on patient recall over the year prior to institution of the CMS changes in a state without such legislation.

## Methods

The Human Subjects Division of the University of Washington gave approval for this research survey. Patients were seen in glaucoma subspecialty clinics of the University of Washington from July 15, 2010 to December 31, 2010. Patients with a diagnosis of glaucoma who were prescribed long-term topical glaucoma therapy in both eyes and who self-administered drops were eligible for this study. The diagnosis of glaucoma was determined by standard ophthalmic examination and ancillary testing by fellowship-trained glaucoma specialists (PPC, RCM), and was based upon characteristic optic nerve appearance regardless of intraocular pressure or visual field findings. We chose to include only patients on bilateral treatment because we reasoned that patients using drops in only one eye on a traditional dosing regimen would be less likely to run out of medications. Because the personnel who administered the survey (CW, KLM) were not able to attend every glaucoma clinic at the University of Washington, not all eligible patients were enrolled. However, when the study-administering personnel were present, all eligible subjects were asked if they wished to participate in a study of eye drop use (no mention was made of eye drop bottle exhaustion). Of the subjects approached to participate in the study, approximately ten refused to participate.

Patients were required to be on a stable, self-administered drop regimen with no changes in their glaucoma therapy during the prior 3 months. After obtaining written informed consent, a short survey was completed (see Appendix) at the time of their office visit with the assistance of an in-person interviewer (CW, KLM). The survey was developed by the authors and was based on clinical experience with patient complaints during glaucoma clinic visits; it was not further validated. Data was collected regarding insurance status, self reported incidence of early eye drop bottle exhaustion, specific drops used, specific drops that were exhausted early, and factors that the patient considered to be responsible for this occurrence. We reviewed the patients’ medical records for additional data, including visual acuity and most recent visual field data, including mean deviation (MD), pattern standard deviation (PSD) and visual field index (VFI). No automated visual field data existed for some eyes with poor visual acuity. For bilateral data, we divided eyes into worse and better eyes, based on visual acuity (visual field data were used if visual acuities were equal in each eye). We considered an eye with Snellen visual acuity of 20/70 or worse to have poor visual acuity, corresponding to the World Health Organization definition of visual impairment [10]. We noted the presence of ophthalmic and medical comorbidities (arthritis was considered separately), and number of other prescription medications. Combination drops (e.g. dorzolamide-timolol) were considered to be one bottle, but two medications. In cases where glaucoma medications included oral medications prescribed for intraocular pressure reduction, these were not considered eyedrops. We did not collect data regarding the brand, fill or bottle type of the medications solicited. Statistical analysis was performed with SPSS 16.0 for Mac (SPSS, Chicago, IL).

## Results

A total of 236 patients completed the survey during the study period. Baseline demographics and characteristics of the study population are presented in Table 1. Sixty patients (25.4%) reported early exhaustion of eye drops at least once in the past year (Table 2). The sole risk factor significantly associated with this level of early bottle exhaustion was poor visual acuity (≤20/70) in the better eye (5/9 vs 55/227; P = .049, Fisher exact test) from any cause.

**Table 1:** Demographics/characteristics of the study population (N = 236)


Age (years)	67.7 ± 14.0
Sex	
Female	119 (50%)
Male	117 (50%)
Visual field indices	
Better eye	MD: −4.2 ± 5.7 dB
	PSD: 4.2 ± 3.4 dB
	VFI: 90 ± 16%
Worse eye	MD: −8.5 ± 8.1 dB
	PSD: 6.5 ± 4.2 dB
	VFI: 77 ± 25%
Duration of treatment	7.9 ± 5.9 yrs
No prescription insurance	23 (9.7%)
Number of eye drop bottles	2.1 ± 0.8
Number of comorbid conditions*	2.3 ± 1.8
Number of prescription medications*	3.3 ± 3.2

Mean ± SD; MD = mean deviation; PSD = pattern standard deviation; VFI = visual field index.

*Other than glaucoma.

**Table 2:** Frequency of reported inadequate eyedrop bottle volume

**Frequency**	**N (%)**
Rarely (1-2x/yr)	29 (12.3)
Sometimes (3-4x/yr)	19 (8.1)
Often (5-7x/yr)	1 (0.4)
Usually (8-11x/yr)	4 (1.7)
Always	7 (3.0)
Total	60 (25.4)

Twelve patients (5.1%) reported that they “often” (5–7 times per year), “usually” (8–11 times per year) or “always” ran out of eye drops before they were able to refill their prescriptions. Risk factors for falling into this group included poor visual acuity (≤20/70) in the worse eye from any cause (P = .015), and worse logMAR visual acuity in the worse eye (P = .043) (Table 3). Reasons for poor visual acuity (≤20/70) for either eye are listed in Table 4. No other significant risk factors were found, specifically including systemic medical problems such as arthritis (N = 47), diabetes mellitus (N = 32), history of cerebrovascular accident (N = 6), Parkinson’s disease (N = 5), essential tremor (N = 7), and any peripheral neuropathy (N = 6) (P > .455; data not shown). No association was found between lack of insurance (N = 12, 5%), or any type of insurance (Medicaid vs. Medicare vs. private), and early bottle exhaustion (data not shown).

**Table 3:** Selected risk factors for early eye drop bottle exhaustion among patients who report often, usually, or always running out of drops early (at least 5 times yearly)

**Factor**	**Yes, runs out**	**No, does not**	**P**
Age (yrs)	71.7 ± 11.3	67.1 ± 14.2	.271*
Years of glaucoma drop use	10.9 ± 7.6	7.8 ± 5.8	.070*
Poor visual acuity (≤20/70)			
Better eye	2/12 (16.7%)	7/224 (3.1%)	.070†
Worse eye	6/46 (13.0%)	6/190 (3.2%)	.015†
logMAR visual acuity			
Better eye	.505 ± 1.03	.087 ± .199	.188*
Worse eye	.881 ± 1.06	.397 ± .787	.043*
Visual field			
Mean deviation (dB), better eye	−6.7 ± 7.6	−4.1 ± 5.6	.131*
Visual Field Index, better eye	81 ± 21	90 ± 16	.198*
Mean deviation, worse eye	−12.0 ± 13.3	−8.4 ± 7.8	.387*
Visual Field Index, worse eye	63 ± 40	78 ± 23	.228*
Number of comorbid conditions (mean ± SD)	2.8 ± 2.0	2.3 ± 1.8	.274*
Number of prescription medications (not including eye drops)	5.3 ± 5.4	3.2 ± 3.0	.228*
Number of bottles	2.1 ± 1.2	2.1 ± 0.8	.962†
Number of eye drops/day	6.2 ± 4.5	6.6 ± 3.8	.235†

*t-test, 2-tail; †Chi square or Fisher Exact test, 2-tail.

**Table 4:** Reasons for reduced visual acuity (≤20/70)

**Primary reason for reduced visual acuity**	**Better eye (N = 9)**	**Worse eye (N = 45)**
Glaucoma (any type)	1 (11%)	11 (24%)
Retinal vein occlusion	0	6 (13%)
Age-related macular degeneration	2 (22%)	5 (11%)
Myopic retinal degeneration	2 (22%)	5 (11%)
Cataract	0	3 (7%)
Amblyopia	1 (11%)	3 (7%)
Retinal detachment	0	3 (7%)
Dry eye disease	1 (11%)	2 (4%)
Posterior uveitis	1 (11%)	2 (4%)
Diabetic retinopathy	0	1 (2%)
Other*	1 (11%)	4 (9%)

*Includes Non-arteritic ischemic optic neuropathy, retinopathy of prematurity, irregular astigmatism after penetrating keratoplasty, macular hole.

Our survey included questions regarding possible reasons that the patient felt contributed to early bottle exhaustion, and 88 total responses were given (some patients listed more than one reason) (Table 5). Of these, the most frequently cited were “insufficient amount in bottle,” “more than one drop comes out,” “can’t hold bottle steady,” “can’t see tip of bottle” and “size of the drops is too large.” Some noted problems with the bottle, specifically that they are not clear, making it difficult to see when the amount of medication is running low. Only 4.5% reported the problem was due to missing the eye with the eye drop.

**Table 5:** Patient questionnaire responses regarding factors contributing to early bottle exhaustion

**Patient-reported reason for early bottle exhaustion**	**N (%)**
More than one drop comes out	27 (30.6)
Insufficient amount in bottle	16 (18.1)
Drop size too large/inconsistent	16 (18.1)
Can’t see tip of bottle	10 (11.4)
Can’t hold bottle steady	8 (9.1)
Misses eye	4 (4.5)
Not sure why	3 (3.4)
Poor vision	2 (2.3)
Can’t see if bottle empty	2 (2.3)

Among medications being used by at least 10% of subjects, the lowest rate of early bottle exhaustion was seen among subjects using brimonidine (4/74, 5.4%), and the highest rate was seen with latanoprost (13/80, 16.3%) (Table 6).

**Table 6:** Number of eyedrops used, exhausted and percentage of drop exhaustion among those with any early eyedrop bottle exhaustion, and those with frequent (≥5 times per year) early eyedrop bottle exhaustion

**Medication**	**N patients using drop**	**N (%) any early exhaustion**	**N (%) frequent early exhaustion**
Timolol/Dorzolamide	89	7 (7.9)	4 (4.5)
Latanoprost	80	13 (16.3)	3 (3.8)
Brimonidine	74	4 (5.4)	1 (1.3)
Timolol	64	10 (14.5)	3 (4.7)
Travoprost	66	10 (15.2)	3 (4.5)
Bimatoprost	41	6 (14.6)	2 (4.9)
Dorzolamide	18	0	0
Pilocarpine	17	2 (11.7)	0
Brinzolamide	16	2 (12.5)	0
Timolol/Brimonidine	5	1 (20.0)	0
Apraclonidine	5	1 (20.0)	0
Betaxolol	5	2 (40.0)	0
Carteolol	5	2 (40.0)	0
Levobunolol	3	0	0

## Discussion

Poor adherence with treatment has been reported as a risk factor for progression to blindness in glaucoma patients [11-13]. This study evaluated the self-reported prevalence of early glaucoma eye drop bottle exhaustion and found 25% of patients reported their bottles did not last until the next allowed refill at least once yearly, and for 5% of patients this occurred on a regular basis (at least 5 times per year). The actual number of patients affected may be even greater, as self-reported non-anonymous studies may underestimate the prevalence of a given problem, due to response bias [14]. Recent CMS changes (at the United States national level) and some state Medicaid changes now allow refills of prescription drugs at 70% of the predicted days of use for patients with prescription drug coverage [9], but this only applies to patients who are Medicare beneficiaries who have enrolled in Part D; in 2011, approximately 13.5 million persons (~28% of those enrolled in Medicare) were not covered by Part D plans [15]. In addition, non-Medicare prescription drug insurance plans are not required to follow the same CMS rules. As such, although there are many potential reasons patients who exhaust their medications earlier than a scheduled refill may not collect new medications, insurance restrictions have been a significant barrier in the United States.

We found an association between poor visual acuity in the worse eye (from any etiology, though in our study about one quarter of worse-affected eyes were damaged by glaucoma) and higher levels of early bottle exhaustion. One possible explanation for this finding may be “increased” adherence with therapy (perhaps better termed over-adherence) as compared to patients with less severe disease. Some investigators have suggested that fear of blindness is a motivator for adherence [16]. Similarly, patients considered glaucoma suspects are less adherent with therapy than patients diagnosed with glaucoma [17,18]. However if such over-adherence leads to early bottle exhaustion, this may only worsen advanced disease. Thus the individuals most affected by early bottle exhaustion are those who need their glaucoma medication the most – those who have limited sight.

In addition, administration of eye drops requires dexterity and hand-eye coordination; good visual acuity and the ability to aim are necessarily interrelated [19]. Indeed, videotaping of glaucoma patients has shown they used an average of 1.4 - 1.8 drops when trying to instill a single eye drop [20,21], which would result in a need to refill medications earlier than expected. A previous study evaluating disease severity and eye-drop administration found that in patients with asymmetric glaucoma, there was a significant association between worse instillation technique and the eye with greater visual field loss [22]. Visual field data was not associated with early bottle exhaustion in our patient population, though this is probably related to the lack of automated visual field testing in eyes with very poor visual acuity. Systemic conditions such as cerebrovascular accident, tremor, Parkinson’s disease, and peripheral neuropathy were poorly represented in our study population, possibly because we excluded patients who did not administer their own drops, and patients severely afflicted by the aforementioned diseases probably must rely on caretakers to administer their eyedrop medications.

Although many of the factors cited by our subjects as reasons for early bottle exhaustion are likely amenable only to personalized instruction or use of an eyedrop dispensing aid, some of them might be amenable to changes in eye drop bottle manufacture: such as colored bottle tips that would allow patients to see the tip of the bottle more easily, clear or translucent bottles so patients could see when they are running low on medication, and development and distribution of aids for eye drop instillation (or greater adoption of existing instillation aids) among those at risk for early bottle exhaustion. Although the reasons we listed on the survey might appear to direct our subjects’ responses, in fact 43% of responses were listed under “other.” Clearly, given the difficulty in eye drop administration, the development of an alternative eye drop delivery system would be welcome.

No association was found between medical insurance status nor prescription drug insurance status, and early eye drop bottle exhaustion, although only a small proportion of our study population was uninsured. While we did not collect data on out-of-pocket drug cost or socioeconomic data for our subjects, the lack of association seen with insurance status or type implies that socioeconomic status may not influence the rate of early bottle exhaustion, which is consistent with other studies that have looked at other sociodemographics such as educational level [6,13,23,24]. A reasonable conclusion, based on our findings and those of other authors, would be that problems with eye drop administration are present across socioeconomic stations and are not related to drug cost or insurance status.

That patients with glaucoma may have poor adherence to topical treatment is not a novel finding. The usual implication is that insufficient medicine is taken despite adequate supply [23-25], although some authors have found that 7-13% of patients overused topical glaucoma drops on a regular basis, 6–7 days per month [5,26]. Our study suggests that providers must also be cognizant of early bottle exhaustion when managing glaucoma patients. Patients with poor vision may have particular difficulty with this issue; unfortunately, these may be the patients that need well-controlled IOP the most. However, treatment should be individualized, since a multitude of factors may interfere with compliance [8]. Medication insurers and pharmacy staff should acknowledge that some patients will have early bottle exhaustion, and should make allowances for early eye drop bottle refills, as is currently permitted under Medicare Part D. Although patients desire personalized instruction on eyedrop administration [27], and while it makes sense that improved training of patients in eyedrop self-administration might also reduce the number of patients that have difficulty with this basic aspect of glaucoma treatment, there is currently no evidence in the literature that supports such a contention [28].

Several limitations of our study must be acknowledged. Our data collection relied solely on patient recall, and was not validated with pharmacy refill data, with observation of eye drop instillation practices or with patient involvement. We studied a relatively small population at a single academic center, and our results may not be generalizable to other populations. Given the small number of patients with higher levels of early bottle exhaustion, our findings on risk factors for early bottle exhaustion should be interpreted with caution. Response bias may have led some patients to minimize or maximize the extent of their difficulties, although we attempted to word our survey carefully in an attempt to avoid such bias. Further investigation of this subject might focus on a larger sample size, prospective measurement via pharmacy refill data, use of eye drop application aids among patients who report difficulty with early eye drop bottle exhaustion and length of time patients must go without medication, as well as coping mechanisms that patients employ to “stretch out” their medication eye drop supply.

## Conclusions

Self-reported early glaucoma bottle exhaustion regularly affected 5% of patients in our glaucoma clinic population, and 25% reported early exhaustion at least once yearly; the main risk factor was poor vision (Snellen acuity of 20/70 or worse) in at least one eye. Our survey was only conducted on experienced eyedrop users who self-adminstered their eyedrops bilaterally, and therefore these results may not be generalizable to a broad patient population with patients suffering from numerous medical issues, or with those inexperienced with eye drop self-administration. However, this pilot study compels further data collection and research, and consideration of early eye drop bottle exhaustion in at-risk glaucoma patients in the clinic.

## Appendix: Eye drop use survey

1. How many different bottles of glaucoma eye drops do you use?

1____ 2____ 3____ 4 or more_____

2. Do you have any kind of insurance plan that helps to pay for your glaucoma eye drops?

a. Yes____

If yes, is this Medicare Part D? Yes____ No____

If yes, name of insurance (if known)__________________

b. No____

3. How often do you think you run out of eye drops before the end of the month, or before your insurance company will allow you to re-order your drops?

a. Never_____

b. Rarely - not more than once or twice per year_____

c. Sometimes - 3 to 4 times per year_____

d. Often - 5 to 7 times per year_____

e. Usually - 8 to 11 times per year_____

f. Always_____

4. Which of your medications usually runs out early?

Name of medication(s)_____________________________

It varies, cannot say____

5. Why do you think you usually run out of drops earlier than you are supposed to? Check all that apply.

I miss my eye because I can’t see the bottle tip well_____

I miss my eye because I can’t hold the bottle steady_____

More than one drop comes out______

The size of the drops is too large______

Other reason (please list)____________________________

## Competing interests

Supported in part by a Departmental Grant from Research to Prevent Blindness, Inc., New York, NY and the University of Washington Glaucoma Research Fund. No sponsor had any role in the design or conduct of this research. None of the authors has financial interest or any form of conflicting interest in any aspect of this manuscript.

## Authors’ contributions

DBM participated in data analysis and preparation of the manuscript. CW participated in patient recruitment and data collection. KLM participated in patient recruitment and data collection. MAS participated in data analysis and preparation of the manuscript. RCM participated in patient recruitment and data collection. PPC participated in study design, patient recruitment, data collection, data analysis and preparation of the manuscript. All authors read and approved the final manuscript.

## Pre-publication history

The pre-publication history for this paper can be accessed here:

http://www.biomedcentral.com/1471-2415/14/79/prepub
